# Cellular Receptors of Amyloid β Oligomers (AβOs) in Alzheimer’s Disease

**DOI:** 10.3390/ijms19071884

**Published:** 2018-06-27

**Authors:** Barbara Mroczko, Magdalena Groblewska, Ala Litman-Zawadzka, Johannes Kornhuber, Piotr Lewczuk

**Affiliations:** 1 Department of Neurodegeneration Diagnostics, Medical University of Białystok, 15-089 Białystok, Poland; ala.litman-zawadzka@umb.edu.pl (A.L.-Z.); piotr.lewczuk@uk-erlangen.de (P.L.); 2 Department of Biochemical Diagnostics, Medical University of Białystok, 15-089 Białystok, Poland; 3 Department of Biochemical Diagnostics, University Hospital in Białystok, 15-276 Białystok, Poland; magdalena.groblewska@umb.edu.pl; 4 Department of Psychiatry and Psychotherapy, Universitätsklinikum Erlangen, and Friedrich-Alexander Universität Erlangen-Nürnberg, 91054 Erlangen, Germany; johannes.kornhuber@uk-erlangen.de

**Keywords:** amyloid-β oligomer, protein aggregation, AβO receptors, Alzheimer’s disease, neurodegeneration

## Abstract

It is estimated that Alzheimer’s disease (AD) affects tens of millions of people, comprising not only suffering patients, but also their relatives and caregivers. AD is one of age-related neurodegenerative diseases (NDs) characterized by progressive synaptic damage and neuronal loss, which result in gradual cognitive impairment leading to dementia. The cause of AD remains still unresolved, despite being studied for more than a century. The hallmark pathological features of this disease are senile plaques within patients’ brain composed of amyloid beta (Aβ) and neurofibrillary tangles (NFTs) of Tau protein. However, the roles of Aβ and Tau in AD pathology are being questioned and other causes of AD are postulated. One of the most interesting theories proposed is the causative role of amyloid β oligomers (AβOs) aggregation in the pathogenesis of AD. Moreover, binding of AβOs to cell membranes is probably mediated by certain proteins on the neuronal cell surface acting as AβO receptors. The aim of our paper is to describe alternative hypotheses of AD etiology, including genetic alterations and the role of misfolded proteins, especially Aβ oligomers, in Alzheimer’s disease. Furthermore, in this review we present various putative cellular AβO receptors related to toxic activity of oligomers.

## 1. Introduction

Alzheimer’s disease (AD) affects tens of millions of people worldwide and estimated number of AD patients would increase to over 130 million by 2050 [1]. AD is a big socio-economical problem because of comprising not only suffering patients, but also their relatives and caregivers. Additionally, current therapeutic strategies provide only palliative, not disease-modifying, agents.

AD belongs to a large group of neurodegenerative diseases (NDs), which include also Parkinson’s disease (PD) and PD-related disorders, prion disease, motor neuron diseases (MND), Huntington’s disease (HD), spinocerebellar ataxia (SCA), spinal muscular atrophy (SMA), and others [2]. Characterized features of NDs are the progressive degeneration and/or death of neuron cells. In AD, this neuronal loss is accompanied by progressive synaptic damage, which results in gradual cognitive impairment and, finally, dementia.

The main histopathological hallmarks of AD are the extracellular plaques within brain tissue consisted of variant forms of amyloid β (Aβ) and neurofibrillary tangles (NFTs) of many forms of phosphorylated Tau proteins (pTau), localized intraneuronally [3]. Primarily, these pathological alterations are seen within medial temporal lobe, whereas in later stages of AD they progress subsequently to brain regions associated with neocortex [4,5]. Formation of Aβ plaques and neuronal cell damage are preceded by reduced synaptic transmission and loss of dendritic spines, which lead to synaptic dysfunction in AD brain. It is estimated that these changes may anticipate the first cognitive decline symptoms even for two decades [6]. Furthermore, declined levels of Aβ42 in cerebrospinal fluid (CSF) and the presence of Aβ plaques in neuroimaging may head other AD-related alterations by many years [7].

## 2. Postulated Hypotheses of AD Etiology

### 2.1. Risk Factors of AD

The risk factors of AD include increasing age, genetic, and vascular factors, smoking, obesity and diabetes [8]. The presence of genetic mutations as the etiological factors of AD has been identified in 1–5% cases [8]. Although most of sporadic AD cases are unrelated to any autosomal-dominant inheritance, certain genetic changes may be linked with a significant risk of AD development. The mutations in presenilin1 (*PS1*), presenilin2 (*PS2*), and amyloid precursor protein (*APP*) genes are associated with the familial Alzheimer’s disease (FAD) (reviewed by Hardy [9]), while the presence of apolipoprotein E (*APOE*) ε4 genotype links to sporadic form of AD [10,11].

### 2.2. Amyloid Hypothesis

The precise etiology of AD remains unknown, despite over century passing from the first report of its symptoms by Alois Alzheimer [12]. Scientific efforts to elucidate AD etiopathogenesis lead to several different, partly complementary hypotheses. The relevant role of Aβ42 aggregation in AD pathogenesis has been disputed for over 25 years. Originally, the imbalance between production and clearance of Aβ42 in the very early stages of AD have been assumed as a causative and initiating factor of this disease. This dyshomeostasis may be the result of the mutations either in APP genes or in genes encoding presenilin, the substrate and enzyme of the reaction that generates Aβ42, respectively. It leads to the presence of Aβ deposits and the damage of the nerve tissue (reviewed by Selkoe [13]). Amyloid hypothesis may by supported by the observation that progressive Aβ deposition is present already in early, preclinical stages of AD and, finally, in all AD patients.

### 2.3. Isoform APOE4

The relationship between impaired amyloid deposition/clearance and genetic risk factors in AD were highlighted. As it was mentioned above, the best known genetic risk factor of AD is the ε4 allele of the APOE [14,15], which is associated with sporadic, late-onset AD. APOE is a polymorphic lipoprotein, with three major gene alleles: *APOE-ε2*, *APOE-ε3*, and *APOE-ε4*. It was shown that these three APOE isoforms bind Aβ differentially and modulate its fibrillogenesis [16,17,18]. The isoform APOE4 is unable to stimulate degradation of Aβ effectively, which results in a decreased brain clearance of Aβ and leads to the accumulation of amyloid deposits in the brain [19]. Moreover, there are more vascular and plaque deposits of Aβ observed in APOE4 carriers than in humans expressing only APOE3 [20]. This observation was also confirmed in genetically engineered mice [10]. Additionally, a quantitative evaluation in transgenic mice bearing human APP and APOE genes has shown decreased Aβ clearance in *APOE4* carriers in comparison with E3 and E2 mice, which was paralleled by the degree of Aβ deposition [21].

### 2.4. Mutations in Presenilins PS1 and PS2 or APP Genes

In the familial form of AD, most cases are related to mutations in genes encoding one of three proteins: presenilins PS1 and PS2 or APP (i.e., the proteases and their substrate for generation of Aβ, respectively) [22]. APP is an essential membrane protein expressed mainly by the synapses and involved in their formation [23] as well as in neural plasticity [24] and iron export [25]. This protein is the precursor molecule that proteolytic processing generates various peptide fragments, including polypeptides of Aβ with 37 to 49 amino acid residues and molecular weight of approximately 4 kDa [26]. Proteolytic cleavage of APP is completed by enzymes of secretase family: α-, β-, and γ-secretase. Whereas APP processing by α- and β-secretase leads to removal of almost entire extracellular domain and produces membrane-anchored C-terminal fragments (reviewed by Zheng [27]), γ-secretase processing of APP results in generation of Aβ fragment [28]. γ-Secretase is a large, multi-subunit enzyme whose catalytic subunit is presenilin, a multi-pass transmembrane protein. The amyloidogenic processing of APP [29] and γ-secretase activity [30] have been associated with lipid rafts within cellular membrane. The role of cholesterol in lipid raft maintenance has been cited as a likely explanation for observations that high cholesterol and APOE-ε4 genotype are the major risk factors for Alzheimer’s disease [31].

Most mutations in the presenilin and APP genes enhance the production of Aβ42 [32] and early-onset deposition of this peptide [33,34,35]. Especially, the mutations in the region of APP molecule corresponding to the Aβ sequence lead to the production of more self-aggregating forms of amyloid [13]. Similarly, different presenilin mutations result in decreased ability of processing of APP by γ-secretase, and consequently increase the relative production of longer, more hydrophobic and more self-aggregating peptides of Aβ [13]. Peptides Aβ42, Aβ43, and longer express high potential of self-aggregation, whereas Aβ40 may rather be anti-amyloidogenic [36]. However, some of the pathogenic presenilin mutations only alter the ratio between Aβ42 and the other peptides of Aβ, especially Aβ40, but do not increase Aβ42 levels [37,38].

### 2.5. Down’s Syndrome

A gene for *APP* is located on chromosome 21. In subjects affected with Down’s syndrome due to the trisomy of this chromosome and possessing three copies of *APP* gene, AD is most likely to develop within the first 40 years of life [39,40]. This duplication of the wild-type *APP* gene leads to early-onset Aβ deposition, which occurs already in the teenagers, is then followed by microgliosis, astrocytosis, and accumulation of NFTs typical for AD. On the contrary, inheritance of a missense mutation in *APP*, that decreases the production and aggregation of Aβ, protects against AD and age-related cognitive decline [41].

### 2.6. Deposition of Misfolded Tau Protein

It was proposed that amyloid cascade is not the only pathway to AD (discussed by [42,43,44]). Although accumulation of Aβ in AD brain is followed by progressive deposition Tau protein, another hypothesis assumes that the abnormalities in the Tau protein initiate the cascade of events in AD [45]. In normal conditions, Tau is a soluble protein that is responsible for the association and stabilization of microtubules. In nerve cells, Tau is typically found in axons, but in the tauopathies, such as AD, progressive supranuclear palsy (PSP), corticobasal degeneration (CBD), or inherited frontotemporal dementia and Parkinsonism linked to chromosome 17 (FTDP-17), this protein is present in an abnormal filamentous form and redistributed to the cell body and neurites [46]. Hyperphosphorylated forms of Tau aggregate in NFTs within the neurons [47]. This results in the disintegration of the microtubule and the destruction of neuronal transport [48], leading to impaired communication between neurons and, finally, their death [49]. This hypothesis may be partially confirmed in a model of AD created by Jack et al. [7], where Tau pathology in brain precedes Aβ deposition in time, but only on at a sub-threshold biomarker detection level. Although some human neuropathological studies suggest that NFTs may occur prior to presence of amyloid plaques (for review see: [13]), it is possible, that such studies might not have searched systematically for diffuse plaques or soluble, oligomeric forms of Aβ in the brain. Moreover, genetic studies prove that Aβ-elevating *APP* mutations lead to downstream alteration and aggregation of wild-type Tau, whereas Tau mutations do not lead to Aβ deposition and amyloid-related dementia. Some researchers suggest that Aβ can trigger AD-type Tau alterations, whereas Tau expression seems to permit certain downstream neuronal consequences of progressive Aβ build-up to arise [50]. This triggering feature is particularly addressed to soluble AβO [51].

### 2.7. Neuroinflammation

The immune system participates also in AD pathogenesis. It was demonstrated that AD patients express decreased levels of naturally-produced antibodies against Aβ when compared with healthy individuals [52]. The inflammatory reaction, oxidative stress and dyshomeostasis of metals metabolism also play an important role in AD pathogenesis [53,54]. It appears that insoluble Aβ deposits are recognized as foreign material and trigger activation of inflammatory response cascade [55,56]. Additionally, inflammation within AD brain may be partially linked with APOE4’s role as an aberrant immunomodulatory factor. The function of macrophages and microglia is regulated by APOE and may vary depending on isoform of this lipoprotein. Especially, APOε4 is associated with an enhanced inflammatory response compared to macrophages not expressing this allele [57]. It was shown that microglia derived from homozygotic mice possessing both alleles *APOε4* demonstrated a pro-inflammatory phenotype, altered cell morphology, increased NOS2 mRNA levels and NO production, as well as higher pro-inflammatory cytokine production compared to microglia derived from *APOε3* mice [58]. This effect was gene dose-dependent and increased with the number of *APOε4* gene alleles. Although the immune aspects of AD draw increasing attention of researchers, the aim of this paper was to concentrate on other aspects of AD, such as soluble Aβ oligomers (AβOs) toxic activity and their putative cellular receptors.

### 2.8. Soluble AβOs Toxicity

Although initiated by Aβ, progression of AD is subsequently complicated and accelerated by other pathological processes, such as Tau pathology or inflammation. It is known that Aβ peptide may be present in various distinct states, including oligomeric forms of Aβ. These oligomeric species are antigenically distinct from monomeric and fibrillar conformations of Aβ peptide [59,60]. Currently, it is supposed that soluble AβOs, but not fibrillar Aβ42 within neuritic plaques, may be the toxic factors acting on a very early stage of AD, perhaps even initiating pathological cascade.

**Figure 1:**
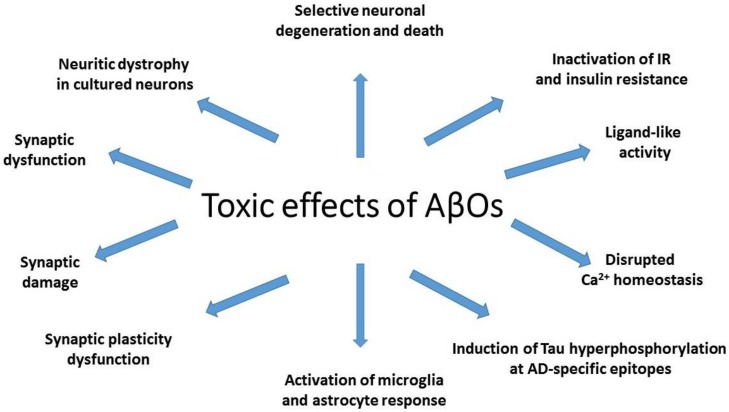
Toxic activity of amyloid β oligomers (AβOs).

Mechanisms of AβO toxicity include synapse loss, the strongest pathological correlate of cognitive deficits in AD (Figure 1). AβO-induced decrease in synapse density is observed already in the earliest stages of AD [61], and the degree of synapse loss is greatest in close proximity of amyloid plaques [62]. The evidence for toxic AβO activity comes from observation that the loss of synapses in AD transgenic animals is correlated with the degree of colocalization of Aβ soluble oligomers with synaptic puncta [63].

Another mechanism of AβO toxicity is oligomers-induced disruption of synaptic transmission. It was demonstrated that soluble AβO could inhibit long-term potentiation (LTP) in mouse hippocampal tissue samples, suggesting that this form of Aβ might be the species triggering loss of synapses and memory impairment in AD [64]. It was also shown in mice model of AD that transgenic animals overexpressing mutant form of human APP exhibited lower density of presynaptic terminals, as well as severe impairments in synaptic transmission in the hippocampus for months before the presence of amyloid plaques [65]. This toxic activity of oligomers was confirmed in the study of Shankar et al., who demonstrated that soluble AβOs isolated from AD patients’ brains decreased number of synapses in animal models of AD, leading to enhanced long-term synaptic depression (LTD) and LTP in regions of brain which are responsible for memory [51].

What is interesting, it seems that intracellular soluble AβOs may be transmitted between neurons using synaptic connections, reaching even distant areas of the brain [66]. It was confirmed in AD mice models, where intracerebral injections of brain extracts from AD or Aβ aggregates induced amyloidogenesis [67]. Although various forms of AβOs may disseminate between neurons in this way, they do not spread between glial cells [68]. It is thought that the self-replication of Aβ and Tau aggregates and their spreading in a prion-like manner may contribute to the progressive nature of AD. Moreover, AβOs can be transferred not only within the brain, from one region to another, but it is supposed that oligomers might be transmitted between people [69].

A limitation of AβOs’ hypothesis may be the fact that oligomers are not homogenous species. Implications for the phenotypic diversity of Aβ in AD include clinical and neuropathological heterogeneity of AD, various distribution and significant differences in the expression of Aβ species in brain tissue, as well as different concentrations of Aβ peptides in CSF of AD patients [69]. AβOs’ molecular weight, morphology and conformation are highly variegated. There are different Aβ forms, from small, dimeric molecules, through trimers, and low molecular weight (LMW) and high molecular weight (HMW) oligomers up to protofibrils and fibrils, which range from relatively small molecules about 4 kDa to assemblies of 100 kDa. Therefore, the precise role of different oligomer species still remains to be elucidated [70,71].

## 3. Cellular Receptors Related to AβOs Activity

Although it is known that extracellular AβOs are able to bind to the surface of neurons, resulting in synaptic dysfunction and neurodegeneration, precise mechanism remains uncertain. It was suggested that AβOs may damage neuronal membranes directly, forming pores, which leads to the ionic dyshomeostasis, especially an increase in intracellular Ca^2+^ levels [72]. It was also proposed that AβOs at high concentration may interact directly with negatively charged phospholipid bilayers, leading to changes in their conductance in non-specific manner [73]. However, it is unclear how such membrane-disrupting activity could explain the selectivity of AβOs for the central nervous system (CNS), especially for the synapses, in AD.

Some other potential mechanisms of AβOs and their targets, including the abnormal activation of signaling pathways, are under extensive investigation [74]. As it was mentioned above, extracellular AβOs accrue generally at synapses, especially at synaptic spines, but detailed specificity of AβOs to particular cells was uncertain. It was shown that in hippocampal cultures Aβ oligomers were bound mostly to neurons, whereas in cortical and cerebellar cultures this binding occurred in a lesser degree [75,76]. It was also demonstrated that both AβOs prepared in vitro and those extracted from AD brain were the ligands targeting cultured mouse hippocampal cells. Soluble AβO isolated from AD patients bound to hippocampal dendrites in cultured mouse neurons with high, “ligand-like” specificity [77]. This specific targeting of neuronal cells is in line with rapid disruption of hippocampal LTP and LTD induced by oligomers [78,79] and with selective neuronal degeneration induced by soluble AβOs seen in brain slice preparations [80].

Several studies postulate various possible receptors involved in the toxicity of AβOs. Binding of AβOs to cell membranes is probably mediated by particular cell surface proteins that act as toxin receptors. This hypothesis could explain various mechanisms of AβOs’ activity, resulting in synaptic dysfunction and neurodegeneration [64].

It is possible that these receptor proteins might be expressed only on certain cells, converting action of AβOs into harmful responses. Moreover, such receptors for extracellular AβO are rather localized at neuronal synapses and should have a high affinity for AβO. These receptors should be also more selective for AβOs than for monomeric or fibrillar Aβ, because monomers of Aβ are present ubiquitously in all individuals, while their levels do not substantially change with disease. Furthermore, putative AβO receptors should have the ability to transduce extracellular triggering factors into certain intracellular changes. It may be achieved either directly or by connection with other active molecules.

There are over 20 candidates for AβO receptors, including glutamate, adrenergic, acetylcholine receptors and others. Unfortunately, no single candidate receptor protein has been shown yet to be responsible for all features of AβO activity. Moreover, the heterogeneity of AβOs results in their diverse affinity as ligands when binding to various putative oligomers’ receptors [81].

### 3.1. Glutamate Receptor NMDAR

Synaptotoxic activity of AβOs includes inappropriate increase of extracellular glutamate concentration and activation of glutamate receptors, which results in rapid impairment of synaptic plasticity [82]. The *N*-methyl-d-aspartate (NMDA) receptor is a glutamate receptor with ion channel activity. It plays a role in controlling of synaptic plasticity and synapse formation, which are responsible for memory function, learning and formation of neuronal networks in CNS [83]. It was suggested that oligomeric Aβ toxicity may involve NMDAR activation, although it remains controversial whether AβOs trigger loss or gain of its function.

Some studies indicate that AβOs initiate impairment of NMDAR activity by removal from the cell surface and triggering of synaptic depression signaling pathways [84,85,86]. By modulation of NMDAR-dependent signaling pathway, AβOs induce reversible synapse loss causing the decrease in spine density [85,87]. Both in vivo and in vitro studies demonstrated that Aβ can disrupt induction of LTP depending on this type receptor [88]. Moreover, activity of this receptor is required for AβOs-induced synaptic depression [87].

On the contrary, other authors demonstrated that AβOs cause an increase of NMDAR receptor function. AβOs induce neuronal oxidative stress through an NMDAR-dependent mechanism. This activity of AβOs is blocked by memantine, an uncompetitive NMDAR antagonist and the drug used to relief AD symptoms [89]. Moreover, it was reported in animal models of AD, that chronic treatment with memantine reduced Aβ deposition in the brain, both insoluble Aβ fibrils and soluble AβOs. Memantine not only inhibited the formation of different types of Aβ aggregates in a concentration-dependent manner, but also led to disaggregation of Aβ42 fibrils [90]. Interestingly, specific antibody to the extracellular domain of the NR1 subunit of NMDARs led also to reduction of AβOs binding to neurons and completely blocked the formation of reactive oxygen species (ROS) [89].

Dysregulation of Ca^2+^ signaling and membrane disturbance, which is thought as a ubiquitous mechanism of soluble AβOs neurotoxicity [91], may also be mediated by activation of NMDAR [92]. AβOs disrupt NMDAR-mediated postsynaptic Ca^2+^ signaling in response to presynaptic stimulation by enhancing the accessibility of extracellular glutamate as well as directly disturbing the NMDARs [93]. This excessive activation of NMDAR leads to disproportionate inflow of Ca^2+^ to neurons and may cause excitotoxicity, a pathological mechanism recognized in some NDs, including AD. It is thought that this aberrant regulation of intracellular Ca^2+^ signaling is an early event in AD, prior to the presence of clinical symptoms. Dysregulation of Ca^2+^ signaling is also believed to be a crucial factor contributing to AD pathogenesis (for review see: [94]).

Moreover, AβOs interfere specifically with several proteins involved in calcium-related signaling pathways, such as calcineurin, which is Ca^2+^-dependent phosphatase, and Ca^2+^/calmodulin-dependent kinase II (CaMKII) [88]. The dynamic balance between these enzymes is presumed to be important for synaptic plasticity. It was demonstrated that LMW AβOs may inhibit CaMKII activity and thus disrupt the equilibrium between above mentioned enzymes [95]. In addition, activation of NMDAR by soluble AβOs involves Ca^2+^-mediated mitochondrial dysfunction as well as decreased CaMKII levels at synapses. This results in dramatic loss of synaptic proteins such as postsynaptic density-95 (PSD-95), dynamin-1 and synaptophysin [96].

NMDAR may also mediate the toxic impact of AβOs on glucose metabolism in neurons. AMP-activated kinase (AMPK) is a key enzyme in energy sensing and metabolic reprogramming under cellular energy restriction, which is associated with some peripheral metabolic diseases, including diabetes. An impaired AMPK function has been linked recently to certain neurological disorders, such as AD [97]. The intracellular ATP levels and AMPK activity were decreased in cultured hippocampal neurons already after short-term exposure to AβOs. This AβO-dependent reduction in AMPK activity is also mediated by glutamate receptors NMDARs, which results in removal of glucose transporters (GLUTs) from the surfaces of hippocampal neurons [97].

### 3.2. Glutamate Receptor AMPAR

The α-amino-3-hydroxy-5-methyl-4-isoxazolepropionic acid receptor (AMPAR) is also a glutamate ionotropic transmembrane receptor that mediates synaptic transmission in CNS. AMPAR is classified as a non-NMDA-type receptor. AMPARs are tetrameric receptors composed of four subunits, labelled as GluA1, GluA2, GluA3, and GluA4. Subunits GluA1 and GluA2 play an important role in synaptic plasticity and LTP [98]. The permeability of AMPAR to Ca^2+^ is related to the GluA2 subunit [99]. Phosphorylation of AMPARs influences ion channel localization and its conductance. Subunit GluA1 has four known phosphorylation sites, but serine 845 (S845) is a residue that plays an essential role in the trafficking of AMPARs toward extrasynaptic sites [100].

Aβ oligomers may cause synaptic dysfunction also by inducing calcineurin-dependent internalization of AMPAR [101]. It was shown in cortical cultures that soluble oligomers of Aβ, but not monomers, mediate the internalization of the AMPAR subunits GluA1/GluA2 by endocytosis [102]. Short-term exposure of hippocampal neurons to AβOs led to noticeable removal of AMPARs from postsynaptic surface and to impaired insertion of this receptor during synaptic potentiation [103]. It is in accordance with the finding that acute exposure of cultured neurons to soluble AβOs induced AMPAR ubiquitination associated with the removal of this receptor from the plasma membrane [104]. Aβ oligomers reduce basal levels of S845 phosphorylation and surface expression of AMPARs affecting AMPAR subunit composition contributing to early synapse dysfunction in a transgenic mouse model of AD [105].

Binding of AβOs to neurons occurs in dendritic spines expressing AMPARs, preferentially GluA2, which is calcium impermeable [106]. Furthermore, pharmacological inhibition of AMPARs leads to reduced AβOs binding. It was demonstrated that the process of rapid internalization of AβOs with surface AMPAR subunits is mediated by calcineurin, whereas inhibition of this phosphatase and AMPARs prevents AβOs-induced synaptic disruption and spine loss [106].

Whereas the role of AMPARs in hippocampal pyramidal neurons containing GluA1 and GluA2 subunits (GluA1/2) has been extensively examined, the importance of AMPAR type having GluA2 and GluA3 (GluA2/3) for synapse physiology was not clear. It was recently revealed that activation of GluA3 AMPARs may constitute novel type of plasticity at synapses [107]. Animal studies shown that in basal conditions GluA2/3 AMPARs are in low-conductance state, shifting to a high-conductance GluA2/3 channels with increased intracellular cyclic AMP (cAMP) levels, which led to synaptic potentiation [107]. It was also indicated that some forms of LTP, such as vestibulo-cerebellar motor learning, may rather require GluA3-AMPARs activation by increasing single-channel conductance mediated by cAMP signaling [108].

Furthermore, the presence of GluA3-containing AMPARs may be also relevant for synaptic and cognitive deficits mediated by AβOs. It was shown in experiments in AD mouse models that all the effects on synapses and memory mediated by soluble oligomeric clusters of Aβ required presence of AMPA receptor subunit GluA3 [109]. Moreover, AβOs blocked synaptic LTP only in neurons expressing this subunit, whereas GluA3-deficient hippocampal neurons were resistant to toxic AβO activity, such as synaptic depression and spine loss. What is important, mice lacking GluA3 subunit did not express memory impairments [109].

Interestingly, abnormal Tau phosphorylation may contribute to AβOs-induced signaling deficits of AMPAR [110]. AβOs led to abnormal Tau distribution in dendritic spines in cultured rodent hippocampal neurons. Aberrant Tau localization was dependent on the phosphorylation of this protein and resulted in early cognitive deficits and synaptic loss [110].

### 3.3. Metabotropic Glutamate Receptor 5 mGluR5

Glutamate is one of the main excitatory neurotransmitters in human CNS and glutamatergic neurotransmission is involved in most aspects of normal human brain function [111]. This neurotransmitter signals through ligand-gated ion channels, such as AMPAR, or through metabotropic glutamate receptors (mGluRs), a family of several G protein-coupled receptors. Two principal signal transduction pathways involving mGluRs are known: cAMP and phosphatidylinositol signal pathways [112].

Metabotropic glutamate receptor 5 (mGluR5) belongs to group I of metabotropic glutamate receptors and activates phospholipase C. This type of receptor has been implicated in a diverse variety of physiological neuronal functions. Moreover, mGluR5 acts postsynaptically as a co-receptor for AβO [113]. Soluble extracellular AβOs bind to lipid-anchored cellular prion protein (PrP^C^) with high affinity and specificity [114,115]. The coexpression of mGluR5 allows PrP^C^-bound AβO for activation of intracellular Fyn kinase, what results in the disruption of synapses [113]. Complexes of AβO with PrP^C^ generate mGluR5-mediated influx of Ca^2+^ in neurons. This influx may be also driven by human AD brain extracts. Aβ peptides also disturb intracellular Ca^2+^ homeostasis. It was demonstrated that Aβ42 oligomers, but not monomers, significantly altered Ca^2+^ release from intracellular stores, which involved mGluR5 and required network activity [116]. In addition, dendritic spine loss is also mediated by AβO-PrP^C^-mGluR5 complexes signaling pathway [113].

### 3.4. Cellular Prion Protein PrP^C^

It seems that significant part of AβO toxicity in AD may be mediated after initial interaction with PrP^C^ on the neuronal surface. In the normal brain, the expression of PrP^C^ is controlled by a feedback loop with amyloid intracellular domain (AICD). PrP^C^ inhibits the activity of β-secretase (β-site APP cleaving enzyme-1, BACE1) [117] as well as AICD production [118], whereas AICD upregulates PrP^C^ expression, which maintains the inhibitory effect of PrP^C^ on BACE1.

This reaction is disrupted in AD, resulting in the binding of increased levels of AβOs to PrP^C^ and disturbed regulation of BACE1 activity. Moreover, PrP^C^ inhibits formation of fibrillar aggregates of Aβ, trapping this peptide in an oligomeric state [119]. Only recently it was demonstrated that PrP^C^ specifically inhibits elongation of Aβ fibrils by binding to the ends of growing polymers [120]. It was shown that this inhibitory effect requires the globular C-terminal domain of PrP^C^, which suggests that PrP^C^ might recognize specific structure that is common to the ends of both oligomeric and fibrillar form of Aβ [120]. This interaction could probably contribute to the neurotoxicity of AβOs.

As it was mentioned above, cellular prion protein PrP^C^ was identified as AβO co-receptor, although the infectious form PrP^Sc^ conformation is not required [115]. PrP^C^ binds Aβ42-oligomers with high affinity and high selectivity. Purified recombinant PrP^C^ interacted directly with AβOs, whereas the binding of synthetic AβOs to neurons decreased in PrP^C^-null mice.

Moreover, PrP^C^ mediates impairment of synaptic plasticity by AβOs [115]. The effects of interaction between PrP^C^ and AβOs on LTP were compared between wild-type and PrP^C^-null mice [115]. It was shown that soluble AβOs reduced LTP in the wild-type mice, but not in the PrP^C^-null mice. It may indicate that PrP^C^ is required to mediate these toxic effects of AβOs. It was also demonstrated that binding onto PrP^C^ induces intracellular Ca^2+^ increase in neurons via the complex PrP^C^-mGluR5, with harmful effects on synaptic transmission [121].

Although additional receptors may contribute to mediation of AβO action, recent investigations indicate that PrP^C^ supposedly plays a primary role (reviewed by Del Rio [122]). PrP^C^ is a glycosylphosphatidylinositol (GPI)-anchored protein. Thus, the mediation of the signal transduction requires the formation of complexes between PrP^C^ and certain transmembrane proteins, such as acetylcholine and glutamate receptors [113,123,124,125,126]. The complex PrP^C^-mGluR5 plays an important role in AβO binding and activity of oligomers in neurons. The signal transduction downstream of AβO-PrP^C^ complexes involves mGluR5, as well as kinases Fyn and Pyk2 [113]. Additionally, after AβOs to binding PrP^C^ and activation of Fyn tyrosine kinase, NMDARs are phosphorylated, which in turn results in altered surface expression, dysregulation of receptor function, excitotoxicity, and dendritic spine retraction [113]. This mechanism is consistent with previous discovery that Fyn is essential for AβO-induced synaptotoxicity [64,127].

Interestingly, it was shown that PrP^c^ also appears to be relevant in α-synucleopathies, such as PD, participating in α-synuclein binding and brain spreading [122].

### 3.5. β_2_-Adrenergic Receptors

The β_2_-adrenergic receptors (β2ARs) are expressed in the brain, especially in regions involved in AD pathogenesis, i.e. hippocampus and cortex [128]. β2ARs play an important role in cognitive functioning. The activation of β2ARs is essential for normal learning and memory [129,130]. Stimulation of β2ARs promotes synaptic LTP in dentate gyrus and hippocampus [131,132,133,134,135,136]. The role of β2AR in memory formation may be confirmed by enhanced expression of β2ARs in dendritic spines [137,138]. β2AR roles in brain are associated with the AMPA-type glutamate receptor [137].

It was demonstrated that β2ARs activation enhances neurogenesis in APP/PS1 mice, a mouse model of AD [139]. Stimulation of these receptors attenuated memory deficits and reduced Aβ accumulation in mouse brain. Moreover, activation of β2ARs enabled the recovery of memory deficits in APP/PS1 mice, enhanced neurogenesis in the dentate gyrus, restored dendritic branches, and spine density in the hippocampus as well as increased the levels of synapse-associated proteins such as synaptophysin, synapsin 1, and PSD-95 [139]. These findings suggest that activation of β2AR protects synapses in this animal model of AD.

Alterations in β2ARs function have been linked to AD, although the results were not consistent. Decreased levels of β2ARs in certain regions of post-mortem human AD brain, such as locus coeruleus and hippocampus, were demonstrated [140,141,142]. Activation of β2ARs resulted in enhanced γ-secretase activity and intensified amyloid plaque formation [143], whereas use of β2AR antagonists conversely attenuated production of Aβ induced by acute stress [144].

On the other side, the administration of ICI, a selective β2AR antagonist, enhanced neuropathological changes, such as increased Aβ plaque burden, as well as accumulation of phosphorylated Tau in a mouse model of AD [145]. Moreover, blockade of β2AR led to cognitive deficits in mice. These results suggest that selective pharmacologic inhibition of β2ARs may have negative effects on AD-like pathology in this animal model of AD. It should be highlighted that the link between β2ARs and AD is likely highly complex.

It was shown that human AβOs, when applied to slices of rodent brain, are able to induce the degradation of β2ARs [146,147]. β2AR levels in hippocampal slices were decreased significantly after exposition to AβOs. Although HMW soluble oligomers of Aβ extracted from AD brain had faint or none cytotoxic activity, they dissociated in alkaline environment to smaller, LMW oligomers (approximately 8–70 kDa). Postincubation LMW were much more bioactive. They induced impaired hippocampal LTP, activated brain microglia and led to decrease in the neuronal levels of β2ARs in mice in vivo [148].

### 3.6. Acetylcholine Receptor α7nAChR

It was shown that Aβ42 binds with high, picomolar affinity to α7 nicotinic acetylcholine receptor (α7nAChR), a neuronal pentameric cation channel [149]. This binding is accompanied with the loss of cholinergic neurons in the brain, resulting in receptor internalization and intracellular accumulation of Aβ [150]. Furthermore, formation of the α7nAChR-Aβ42 complex was suppressed by shorter chains of Aβ (12–28), indicating that this sequence region contains the binding epitope of amyloid [149].

It was also demonstrated in immunohistochemical studies on human sporadic AD brains that α7nAChR is present in neuritic plaques and co-localizes with Aβ in individual cortical neurons [149]. Moreover, the presence of intracellular AβOs was shown in human cholinergic basal forebrain neurons, suggesting the role of amyloid oligomers in cholinergic deficiency [151]. It was confirmed in a triple-transgenic mouse model of AD, where loss of the α7nAChRs was restricted to brain regions that accumulate Aβ intraneuronally [152].

The loss of α7nAChR enhances AβOs accumulation in a mouse model of AD, exacerbating early-stage cognitive decline and septo-hippocampal pathology [153]. In α7nAChR-null mice crossed with those transgenic for mutant human APP, a neurodegeneration in hippocampus and cognitive decline were found already in early, pre-plaque stage of AD. These changes were associated with the appearance of a small, dodecameric form of AβO [153]. Presented findings suggest that α7nAChR plays a protective role for AβOs toxicity. What is more, restoring LTP impaired by AβOs is possible by using a selective neuronal nicotinic receptor partial agonist SSR180711, which completely rescued both early and late LTP impaired by Aβ42 oligomers [154].

### 3.7. Insulin Receptor

A pathophysiological connection between AD and diabetes was confirmed in numerous studies (reviewed by de Felice [155]). An increasing body of evidence indicates that AD may be called a “brain-specific form of diabetes” or “type 3 diabetes” [156,157]. Both diseases are characterized by key pathological features such as insulin resistance, inflammation, and altered metabolism. Diabetic pathophysiology includes reduction in brain insulin signaling, decreased levels of brain insulin, and elevated levels of glucose.

Toxic activity of AβOs may be also linked with impaired insulin signaling and brain insulin resistance, which lead to elevated Aβ production and reduced AβO clearance, resulting in oligomers’ deposits in the brain and neuronal damage [158]. Furthermore, it was shown that signal transduction by neuronal insulin receptors (IRs) is highly sensitive to soluble AβO. AβOs themselves can influence IRs and decrease brain insulin signaling [158]. In addition, AβOs bind to neuronal IRs and affect its insulin-induced autophosphorylation, preventing activation of specific kinases required for LTP [159]. In cultures of mature hippocampal neurons, soluble oligomers caused a rapid, substantial loss of surface IRs, especially on dendrites bound by AβOs [106].

### 3.8. p75 Neurotrophin Receptor p75NTR

It was suggested that AβOs may induce neuronal death via nerve growth factor (NGF) receptor by alteration of NGF-mediated signaling in cultured cells [75,160]. NGF mediates cell loss through low-affinity receptor for nerve growth factor, also called p75 neurotrophin receptor (p75NTR), which belongs to the tumor necrosis factor (TNF) receptor superfamily [74]. Precisely, toxic effects of Aβ mediated by p75NTR depend on a death domain in the cytoplasmic part of this receptor molecule [161,162]. Synapse targeting of AβOs involves activation of p75NTR. AβOs, together with PrPC, bind at the membrane receptors, forming annular amyloid pores and ion channels to induce aberrant cytoskeletal changes in dendritic spines [96].

In the mouse hippocampus, the expression of p75NTR induced by AβOs involves insulin-like growth factor 1 receptor (IGF-1R) signaling [163]. Significantly elevated hippocampal expression of membrane-associated p75NTR protein was shown in transgenic AD mice and was associated with the age-dependent increase of Aβ42 levels. Moreover, it was demonstrated that microinjections of AβOs induced p75NTR expression in the hippocampus through phosphorylation of IGF-1R, whereas co-administration of IGF-1R inhibitor blocked AβOs-induced overexpression of p75NTR [163].

Conflicting evidence exists regarding the role of p75NTR in AD, especially against toxicity of AβOs. Although an important role of p75NTR in Aβ metabolism and Aβ-mediated neurodegeneration in AD brains was shown, this protein also promotes the differentiation and survival of vertebrate neurons [164]. Furthermore, a conflicting role of p75NTR in the cytotoxic function of Aβ depends on the different state of this peptide. In fact, the neurotoxicity of the two forms of Aβ, insoluble fibrillar or soluble oligomeric form, occurs with different mechanisms. Primarily, it was proved that the expression of p75NTR is required for cell death by fibrillar form of Aβ [165]. Interestingly, the toxicity of fibrillar Aβ species is strictly dependent on p75NTR, whereas neurotoxicity of soluble AβOs is independent of p75NTR and is even decreased by the presence of this receptor. Moreover, the expression of p75NTR protects against the neurotoxicity of oligomers [166]. This protective effect results from an active function of the juxtamembrane sequence of the cytoplasmic region of p75NTR and is mediated by phosphatidylinositide 3-kinase (PI3K) activity [166]. These results suggest that p75NTR might have diverse functions in cell death and survival.

### 3.9. Immunoglobulin and Immunoglobulin-Like Receptors

Human leukocyte immunoglobulin-like receptor B2 (LilrB2) belongs to the subfamily B class of leukocyte immunoglobulin-like receptors (LIR) expressed on immune cells. LilrB2 inhibits stimulation of an immune response, controls inflammatory responses and cytotoxicity, and limits autoreactivity of immune system. LilrB2 binds to major histocompatibility complex (MHC) class I molecules on antigen-presenting cells. It was indicated that MHC class I molecules have additional functions in CNS [167]. Furthermore, numerous MHC class I antigens and their binding partners are found to be expressed in CNS neurons and might be involved in activity-dependent synaptic plasticity [167]. LilrB2 also participates in the process of synaptic plasticity and neurite growth in CNS [168].

Murine homolog of LilrB2, paired immunoglobulin-like receptor B (PirB), is an immune inhibitory receptor, primarily identified in mouse immune cells [169]. Expression of PirB is also observed in subsets of neurons throughout mouse brain. In addition, PirB participates in the inhibition of axonal regeneration [170,171]. It was also suggested that PirB plays an important role in age-related hippocampal aging, synaptic loss and neurotransmitter release, which causes cognitive dysfunction associated with AD [172].

Importantly, murine PirB and its human orthologue LilrB2 are thought to be nanomolar affinity receptors for Aβ oligomers [168]. The interaction between AβOs and PirB/LilrB2 are mediated by the first two extracellular immunoglobulin domains of the receptors [168]. PirB regulates synaptic plasticity, affecting hippocampal LTP, which contributes to Aβ-induced deficits of memory in a mouse model of AD [168]. Moreover, high PirB expression is required for the harmful effect of AβOs on hippocampal formation [168]. In double transgenic APP/PS1 mice, ocular dominance plasticity (ODP) was defective during the very early period of synaptic plasticity development [173]. This observation is in contrast with enhanced ODP during the critical period and in adult mice lacking PirB [174]. It suggests that impaired ODP is one of the earliest Aβ-induced deficits in a mouse model of AD. While Aβ42 oligomers robustly bound to LilrB2-expressing heterologous cells, only a minimal binding of monomeric Aβ42 to LilrB2 was observed [168], which suggests selectivity of AβOs reaction with this receptor. Although similar levels of LilrB2 were detected either in human AD brains or in specimens from non-demented adults, downstream signaling was altered in AD specimens.

It was suggested that FcγRIIb (Fragment crystallizable gamma receptor II b) may also play a role as a AβOs receptor, mediating neurodegeneration and toxic activity of oligomers [175]. FcγRIIb belongs to family of Fc-gamma receptors (FcγR) which have a binding specificity for the Fc (Fragment, crystallizable) region of immunoglobulin gamma (IgG) [176]. They are present on the surface of B lymphocytes, dendritic cells, natural killer cells, macrophages, granulocytes, mast cells, and other cells of the immune system. Additionally, all of the Fcγ receptors (FcγR) belong to the immunoglobulin superfamily and differ in their affinities for IgG due to variegated molecular structure of different IgG subclasses.

It was demonstrated that FcγRIIb is an important factor contributing to the AβOs’ neurotoxicity and memory impairment. This protein was significantly upregulated in the hippocampus of AD brains and neuronal cells exposed to synthetic Aβ [175]. Soluble Aβ oligomers interacted with FcγRIIb both in vitro and in AD brains, whereas inhibition of that interaction blocked neurotoxicity of synthetic AβO. Moreover, in mouse model of AD, genetic depletion of FcγRIIb rescued memory impairments and prevented AβO-induced inhibition of LTP, which supports an idea that this receptor could play an essential role in Aβ-mediated neuronal dysfunction [175].

### 3.10. Triggering Receptor Expressed on Myeloid Cells 2 TREM2

Triggering receptor expressed on myeloid cells 2 (TREM2) is a transmembrane-glycoprotein receptor that is present on the surface of immune cells of myeloid origin [177]. As a lipid-sensing activating receptor, TREM2 binds to phospholipids, apolipoproteins, and lipoproteins through its immunoglobulin-like domain [178]. Moreover, TREM2 interacts with TYRO protein tyrosine kinase-binding protein, also known as DNAX-activating protein of 12 kDa (DAP12), which is an adapter protein for this receptor. In the brain, this interaction triggers the phagocytosis of apoptotic neurons and Aβ peptide in microglia with no inflammatory effects [179].

It was shown that certain coding variants in *TREM2* gene are associated with increased risk for AD [180], which suggests that immune cell dysfunction may also play a role in AD pathogenesis. Normal proteolytic maturation of full-length TREM2 at the plasma membrane is disturbed in mutations of *TREM2* gene, resulting in impaired phagocytosis, which may contribute to the pathogenesis of AD [179].

In normal conditions, TREM2 directly binds to AβOs with nanomolar affinity. Only recently, it was demonstrated that in AD-associated TREM2 mutations this binding is reduced [181]. Moreover, the degradation of Aβ in primary microglial culture and mouse brain was impaired in TREM2 deficiency, resulting in microglial depolarization, induction of K^+^ current into cells as well as increased cytokine expression and secretion, cells migration, proliferation, apoptosis, and morphological changes are dependent on TREM2 [182]. Additionally, TREM2-DAP12 interaction was enhanced by AβOs, which demonstrates that TREM2 may act as a microglial AβO receptor that mediates physiological and AD-related pathological effects [181].

### 3.11. Tyrosine Kinase Ephrin Receptors Eph4A and EphB2

It was suggested that the tyrosine kinase Eph receptors may also play a role in AβOs-induced synaptotoxicity [183]. Eph receptors were named after the cell line from which the cDNA was first isolated, erythropoietin-producing hepatocellular carcinoma. Based on the affinities for binding ligands and similarity of extracellular domain sequences, they are divided into two functionally different groups: EphA and EphB. There are nine EphA receptors (1–9), which bind to ephrin-A ligands (ephrin-A 1–5), proteins anchored to the cell membrane by GPI motif, whereas five EphB receptors (1–5) bind to ephrin-B ligands (ephrin-B 1–3) with a transmembrane domain and a short cytoplasmic region [184].

Eph receptors and their ligands play a key role in the physiological functioning, development and maturation of nervous system [183]. Since Eph ligands and receptors are both membrane-bound proteins, the Eph/ephrin binding and activation of their intracellular signalling pathways may occur via direct cell-to-cell interactions only. In particular, their presence both in pre- and postsynaptic regions is necessary for the development and stabilization of synapses, although EphB and EphA play opposite roles [185].

It was shown, that EphB promotes morphogenesis and growth of dendritic spines, whereas their development is aberrant in the absence of these receptors in hippocampal neurons [186]. Moreover, the formation of synapses is induced by activation of EphB2 receptor via interaction with NMDAR [187].

EphB receptors are also important factors in the pathophysiology of AD and other neuropathologies [188]. It was demonstrated that EphB2 levels in the membrane of hippocampal neurons were decreased after short term treatment of AβOs [189], which could be a result of NMDAR activation [190]. It was also shown that AβOs binding to the fibronectin (FN) type III repeat domain of EphB2 triggers to endocytosis of this receptor and its degradation in the proteasome [191]. The results of EphB2 degradation are the impairment of NMDAR functioning and cognitive deficits. What is interesting, these interaction sites of the EphB2 FN domain with AβOs may be blocked by a small, 10 amino acids length peptide Pep63, which rescued memory deficits in mouse model of AD [192]. These results suggest that inhibition of EphB2-AβOs interactions may be a promising strategy for AD treatment. Furthermore, it was shown induction of the EphB2 expression in the dentate gyrus prevented the cognitive deficits and LTP impairments in mice model of AD [106]. On the other hand, the decrease of AMPAR and NMDAR levels induced by AβOs may be prevented by overexpression of EphB2. This protective effect could be directly related to PDZ-binding motif of EphB2 [191,193,194].

EphA receptors bind membrane-bound ephrinA family ligands residing on adjacent cells, leading to contact-dependent bidirectional signalling [195]. EphA4 receptor plays also an important role in the regulation of synapses functioning in the nervous system and in the repairment after injury, preventing axonal regeneration as well as in the angiogenesis and formation of vessels within central nervous system [183].

EphA4 mediates dendritic spine remodeling and contributes to homeostatic plasticity through the regulation of AMPAR levels [195,196,197]. Activation of EphA4 receptor induces a decrease in the strength of the excitatory synapse as well as reduction of spine length and density in hippocampal slices [195]. This receptor is also associated with the loss of dendritic spines, their retraction and growth cone collapse [195]. It was confirmed in EphA4-knockout mice, which expressed disorganized, longer, and more numerous spines than wild-type mice [183].

Moreover, EphA receptors seems to be key player in the pathophysiology of AD and other neuropathologies, such as motor neuron degeneration in amyotrophic lateral sclerosis (ALS) [198,199]. It was shown in AD brains, that hippocampal distribution of EphA4 was co-localized with neuritic plaques already at early stages (Braak stage II), which suggests that EphA4 may contribute to synaptic dysfunction [200]. Additionally, it was demonstrated that levels of EphA4 mRNA in synaptoneurosomes from AD patients were twofold higher than in non-demented controls [201].

Furthermore, AβOs aberrantly activate EphA4 leading to dendritic spine elimination, whereas blockade or absence of this receptor in hippocampal neurons prevents synaptic loss [202,203]. This AβOs-EphA4 axis involves c-Abl tyrosine kinase activation by AβOs in dendritic spines of cultured hippocampal neurons, which is required for AβOs-induced synaptic loss [183,203].

### 3.12. Receptor for Advanced Glycation Endproducts RAGE

RAGE is a small, 35 kDa transmembrane protein, which belongs to the immunoglobulin superfamily and plays a role in innate immunity. RAGE is composed of three extracellular Ig-like domains (Vd, C1d, C2d), with a single transmembrane domain, and a short cytoplasmic tail [204]. This receptor is described as a “pattern recognition” receptor because of its ability to recognize common structural motifs. RAGE is able to bind multiple ligands, such as advanced glycation endproducts (AGE), glycans and glycoproteins, as well as chromatin protein high mobility group box 1 protein (HMGB-1), calprotectin and S100B [205]. After stimulation, RAGE activates certain pro-inflammatory genes, which mediate Aβ-induced oxidative stress.

Moreover, activation of RAGE results in continuous instigation of nuclear factor kappa-light-chain-enhancer of activated B cells (NF-κB) [206,207]. On the other hand, RAGE itself is upregulated by NF-κB, thus creating RAGE/NF-κB axis. This forms a positive feed-back loop leading to chronic inflammation, altered micro- and macrovasculature, and tissue damage, pathological events observed also in AD.

Expression of RAGE is increased in the AD brain. Moreover, enhanced levels of RAGE ligands were observed in a range of inflammatory diseases, atherosclerosis, diabetes and cancer as well as in AD, which suggests a causative role of this receptor in inflammatory chronic state [207]. RAGE was also identified as one of the cell-surface binding sites for Aβ peptide at the plasma membrane of neurons, microglial cells, and endothelial cells of the vessel wall [206].

Furthermore, RAGE participates in the clearance of Aβ. The level of Aβ peptides as well as other substances in the brain is not only a result of specific equilibrium between their synthesis and degradation, but also depends on the transport into the brain from blood and efflux from the brain into blood through blood-brain barrier (BBB). Both in normal aging and in AD, the rate of CSF reabsorption into the blood, known as bulk flow, is also impaired. The main receptors for the transport of Aβ across BBB are RAGE and low-density lipoprotein receptor related protein-1 (LRP1). RAGE is responsible for Aβ influx, whereas LRP1 is the main receptor controlling the efflux across the BBB to the plasma [208]. Soluble form of LRP1 sequesters 70–90% of plasma Aβ peptides in normal conditions. In AD, this Aβ clearance is disturbed [206]. Both in AD mouse models and in AD patients, the brain endothelial expression of RAGE is elevated, whereas plasma levels of sLRP1 and its Aβ-binding capacity are decreased, leading to increase in free Aβ fraction in plasma [209].

Interestingly, distinct regions of RAGE are induced by different Aβ conformations in AD-related apoptosis [204]. It was demonstrated that anti-RAGE antibodies significantly improved survival of cortical rat neurons and RAGE-expressing cells exposed to either AβOs or aggregated Aβ. Moreover, the use of site-specific antibodies against domain Vd of this receptor prevented AβOs-induced neurotoxicity, whereas blockade of the apoptosis induced by aggregated Aβ required neutralization of C1d domain of RAGE [204].

### 3.13. Megalin Receptor

Megalin, also known as glycoprotein 330 (gp330) or low-density lipoprotein-related protein 2 (LRP2), is a large, approximately 600 kDa protein, which is a multiligand binding receptor expressed in the plasma membrane of epithelial cells [210]. In CNS, megalin is present in choroid plexus epithelium and ependymal cells covering the brain ventricles. LRP2 is a member of a family of receptors with structural similarities to the low-density lipoprotein receptor (LDLR). Megalin mediates endocytosis of its ligands, which results in the degradation in lysosomes or transcytosis [211]. This receptor has been shown to interact with various ligands, vitamin-binding proteins, carrier proteins, lipoproteins, hormones and hormone precursors, as well as drugs and toxins [212]. Moreover, this receptor has also functions in cellular communication and signal transduction, with PSD-95 as an interaction partner [213].

LRP2 is also an endocytic receptor for apolipoprotein J (ApoJ)/clusterin involved in rapid receptor-mediated uptake or bidirectional exchanges of soluble Aβ across BBB [214]. ApoJ has been revealed to be the major protein binding Aβ in CSF. Megalin mediates cellular uptake and transport of ApoJ alone and ApoJ complexed with Aβ-40, the most abundant amyloid isoform found in Aβ deposits of the blood vessels, from the periphery into the brain at the cerebral vascular endothelium and choroid epithelium [215]. This interaction of ApoJ-Aβ complex with megalin is thought to be another, besides RAGE and LRP1, mechanism preventing pathological accumulation of Aβ [216]. It was shown that Aβ alone did not bind directly to LRP-2, whereas complexes of Aβ-40 with apoJ were able to react with megalin. Moreover, ApoJ/Aβ binding interaction was blocked polyclonal anti-megalin antibodies, which supports the role of LRP-2 as a mediator of the clearance of ApoJ/Aβ complex from CSF and in the regulation of Aβ accumulation [216].

### 3.14. Nuclear Receptors

Nuclear receptors (NRs) constitute a class of proteins that mediate certain, relatively small, molecules pathways, thus controlling the development, homeostasis, and various metabolic processes. There are currently 48 nuclear receptors known in the human genome, most of them have identified specific ligands [217]. NRs are involved in the synthesis and metabolism of steroid and thyroid hormones as well as and various other lipid-soluble signals, including retinoic acid, oxysterols, vitamin D, cholesterol, lipids, and bile acids or thyroid hormone [217].

Moreover, many of NRs, but not all, directly bind to signalling molecules. These molecules are small and have lipophilic character, therefore they can easily enter the target cell. Thus, unlike described above membrane-bound receptors, NRs are intracellular proteins which are capable of direct binding to DNA, thus controlling the expression of adjacent genes, which is their unique property that differentiates them from other classes of receptors. Because of this ability, NRs are classified as transcription factors [218].

The nuclear receptor superfamily may be divided according to their amino acid sequence similarities in six subfamilies, which are thyroid hormone receptor-like, retinoid x receptor-like, estrogen receptor-like, nerve growth factor IB-like, steroidogenic factor-like and germ cell nuclear factor-like [217]. Moreover, some NRs require heterodimerization with retinoid X receptor (RXR) [217]. Furthermore, NRs-ligands interactions are characterized by certain redundancy: ligands are nonselective for particular receptors, which also share their transcriptional targets, serving as transcriptional inducers of one another. However, several NRs remain with unknown ligands and are described as “orphan receptors” [219].

All NRs are conservative and similar in their general structure, which includes a ligand-binding/dimerization domain (LBD) and DNA-binding/weak dimerization domain (DBD) as well as at least one N-terminal ligand-independent transactivation region, referred to as AF-1 for activation function 1 (or the A/B domain), and a ligand-dependent transcription region AF-2. To bind DNA, AF-2 may form complexes with co-regulatory proteins that can act as co-activators or co-repressors. AF-2 co-activators regulate histone acetyltransferase activity, whereas its co-repressors control histone deacetylase activity [218].

Certain NRs are also linked with AD pathology as well as with AβOs toxicity. One of these receptors is vitamin D receptor (VDR) that is broadly expressed in brain and regulates many genes. VDR mediates action of Vitamin D (1,25-(OH)_2_D_3_), an important neurosteroid, which plays key role in the brain functioning, such as calcium signaling, cell proliferation and differentiation. Vitamin D is also a neurotrophic factor that regulates neurotransmission and synaptic plasticity. It was revealed recently, that Vitamin D treatment results in significant increase of LRP1 expression both in-vivo and in-vitro studies [220]. Moreover, it was suggested that VDR deficiency/inhibition can be a potential risk factor for AD [221]. It was shown that Vitamin D may be also involved in Aβ clearance. 1,25-(OH)_2_D_3_ increases transport of Aβ across the BBB by regulating expression of amyloid transporters, such as LRP-1, via its nuclear receptor VDR only, or by binding heterodimeric complexes of VDR with RXR [222].

### 3.15. Sirtuin

Sirtuin 1 (SIRT 1), one of NRs that has recently emerged as a crucial protein that may play protective roles in AD and other NDs, including PD and MND (for review see [223]). SIRT1 belongs to the family of sirtuins (Sir2, silent information regulator 2 protein) that was shown to regulate lifespan in lower organisms and affect diseases of aging in mammals. SIRT1 is a nicotinamide adenine dinucleotide (NAD^+^)-dependent histone deacetylase involved in calorie restriction (CR) (reviewed in [224]). Calorie restriction promotes mammalian cell survival by inducing the SIRT1 deacetylase. As it was mentioned above, it was proposed that AD may be described as new form of diabetes or “type 3 diabetes”. The resistance to insulin and insulin-like growth factor are thought to be crucial for the progression of AD [225]. SIRT1 deficiency is also ascribed to be responsible for the increased risk of insulin resistance, obesity and diabetes, including type 3 diabetes, whereas low-calorie diet and nutrition reverse type 3 diabetes and accelerated aging linked to global chronic diseases [226].

SIRT1 is involved in neurodevelopment, including axon elongation, neurite outgrowth and dendritic branching [223]. Furthermore, this NR is also essential for normal cognitive function and synaptic plasticity. It was demonstrated that SIRT1 attenuates amyloidogenic processing of APP by increasing α-secretase activity via SIRT1-coupled retinoic acid receptor-β (RARβ) activation [227]. Upregulation of α-secretase shifts APP processing to non-amyloidogenic cleavage of APP and reduces the pathological accumulation of the toxic Aβ species that results from β- and γ-secretase activity. It may be confirmed by the fact that a significant decrease in SIRT1 level, both mRNA and protein, was observed in the cortex of AD patients [228]. SIRT1 reduction paralleled tau accumulation in the AD brain and may be closely associated with deposition of Aβ in the cerebral cortex of patients with AD.

Although it is difficult to determine when exactly SIRT1 loss occurs in AD, it was suggested that it may be rather a relatively late event. A significant correlation was observed between SIRT1 and the duration of AD symptoms, accumulation of tau, as well as Aβ42 deposition [228]. Furthermore, it seems that AβOs toxicity and their binding to AβO receptors are rather primary toxic effects, than secondary to NRs disturbances with consecutive AβOs receptor interactions.

## 4. Conclusions

Since its first description over hundred years ago, Alzheimer’s disease is one of the diseases in modern biomedicine that have garnered most scientific attention. Within these 100 years there have emerged various hypotheses in order to explain underlying pathology. The dominant model of AD pathogenesis is amyloid hypothesis, although its details were changing over this time, indicating increasing role of oligomeric amyloid beta species as the main toxic factors leading to damage of neurons and loss of synapses. Recent studies have identified that soluble Aβ oligomers interact with certain receptor proteins.

In conclusion, a variety of specific receptors could be responsible for mediating the synaptotoxicity caused by AβOs in AD (Table 1). The AβO-associated receptors include ionotropic and metabotropic glutamate receptors NMDAR, AMPAR, and mGluR, their co-receptor—cellular prion protein PrPc, ephrin receptors EphB2 and EphA4, RAGE, immunoglobulin and immunoglobulin-like receptors FcγRIIB and PirB/LiL2R, neurotrophin receptor p75NTR, β-adrenergic as well as acetylcholine receptors a7nAChRs. Despite over twenty various protein receptors proposed within over twenty years of amyloid hypothesis, no single candidate receptor has been revealed to be necessary and sufficient to account for all features of AβO toxic activity. Taken together, it seems that among this abundancy glutamate and Eph receptors could explain most of the pathophysiological defects and structural changes observed in central nervous system. However, further studies are needed to determine the relevance and contribution of each of these molecules to the pathogenesis of this disease.

## Figures and Tables

**Table 1:** Cellular receptors related to amyloid β oligomer (AβO) activity.

Name of the Receptor	Abbreviation	References
*N*-Methyl-d-aspartate receptor	NMDAR	[83,84,85,86,87,88,89,94,96,97]
α-Amino-3-hydroxy-5-methyl-4-isoxazolepropionic acid receptor	AMPAR	[100,101,103,104,105,106,107,108,109,110]
Metabotropic glutamate receptor	mGluR	[112,113,116]
Cellular prion protein	PrP^c^	[113,115,120,121,122]
β_2_-Adrenergic receptor	β2AR	[128,140,141,142,143,144,145,146,147,148]
α7 nicotinic acetylcholine receptor	α7nAChR	[149,150,151,152,153,154]
Insulin receptor	IR	[158,159]
p75 neutrotrophin receptor	p75NTR	[96,163,164,165,166]
Human leukocyte immunoglobulin-like receptor B2	LilrB2	[167,168]
Paired immunoglobulin-like receptor B	PirB	[168,172]
Fragment crystallizable gamma receptor II b	FcγRIIb	[175,176]
Triggering receptor expressed on myeloid cells 2	TREM2	[181,182]
Tyrosine kinase ephrin type-A receptor 4	Eph4A	[183,202,203]
Tyrosine kinase ephrin type-B receptor 2	EphB2	[183,188,189,190,191,192,193,194]
Receptor for advanced glycation endproducts	RAGE	[204,206,207,208,209]
Megalin (glycoprotein 330, low density lipoprotein-related protein 2)	gp330, LRP2	[210,211,212,213,214,215,216]

## Abbreviations

AβOs	Amyloid β oligomers
AD	Alzheimer’s disease
NDs	Neurodegenerative diseases
Aβ	Amyloid beta
NFTs	Neurofibrillary tangles
PD	Parkinson’s disease
MND	Motor neuron diseases
HD	Huntington’s disease
SCA	Spinocerebellar ataxia
SMA	Spinal muscular atrophy
pTau	Tau protein
CSF	Cerebrospinal fluid
PS	Presenilin
APP	Amyloid precursor protein
FAD	Familial Alzheimer’s disease
APOE	Apolipoprotein E
PSP	Progressive supranuclear palsy
CBD	Corticobasal degeneration
FTDP-17	Frontotemporal dementia and Parkinsonism linked to chromosome 17
LTP	Long-term potentiation
LTD	Long-term synaptic depression
LMW	Low molecular weight
HMW	High molecular weight
CNS	Central nervous system
NMDAR	*N*-methyl-d-aspartate receptor
ROS	reactive oxygen species
CaMKII	Ca^2+/^calmodulin-dependent kinase II
PSD-95	Postsynaptic density-95
AMPK	AMP-activated kinase
GLUTs	Glucose transporters
AMPAR	α-amino-3-hydroxy-5-methyl-4-isoxazolepropionic acid receptor
mGluRs	Metabotropic glutamate receptors
PrPC	Cellular prion protein
AICD	Amyloid intracellular domain
BACE1	β-site APP cleaving enzyme-1
GPI	Glycosylphosphatidylinositol
β2ARs	β2-adrenergic receptors
α7nAChR	α7 nicotinic acetylcholine receptor
IR	Insulin receptor
NGF	Nerve growth factor
p75NTR	p75 neurotrophin receptor
TNF	Tumor necrosis factor
IGF-1R	Insulin-like growth factor 1 receptor
PI3K	Phosphatidylinositide 3-kinase
LilrB2	Leukocyte immunoglobulin-like receptor B2
LIR	Leukocyte immunoglobulin-like receptors
MHC	Major histocompatibility complex
PirB	Paired immunoglobulin-like receptor B
ODP	Ocular dominance plasticity
FcγR	Fc-gamma receptors
IgG	Immunoglobulin gamma
TREM2	Triggering receptor expressed on myeloid cells 2
DAP12	DNAX-activating protein of 12 kDa
Eph	Erythropoietin-producing hepatocellular carcinoma
FN	Fibronectin
RAGE	Receptor for advanced glycation endproducts
LRP	Low density lipoprotein-related protein
RARβ	Retinoic acid receptor-β
HMGB-1	High mobility group box 1 protein
AGE	Advanced glycation endproducts
NF-κB	Nuclear factor kappa-light-chain-enhancer of activated B cells
gp330	Glycoprotein 330
LDLR	Low density lipoprotein receptor
NR	Nuclear receptor
VDR	Vitamin D receptor
LBD	Ligand-binding/dimerization domain
DBD	DNA-binding/weak dimerization domain
AF-1	Activation function 1
RXR	Retinoid X receptor
SIRT 1	Sirtuin 1
